# Herpes Simplex Virus Hepatitis in Patients Requiring Intensive Care Unit Admission: A Retrospective, Multicenter, Observational Study

**DOI:** 10.1093/ofid/ofad484

**Published:** 2023-10-03

**Authors:** Thomas Frapard, Giuliana Amaddeo, Maxens Decavele, Paer-Selim Abback, Antoine Gaillet, Charlotte Bouzbib, Claire Vanlemmens, Romy Younan, Emmanuel Canet, Anne Sophie Moreau, Mathilde Neuville, Elie Azoulay, Alexandre Sitbon, Djamel Mokart, Sylvie Radenne, Armand Abergel, Céline Guichon, Olivier Roux, Agnes Bonadona, Armand Mekontso Dessap, Audrey De Jong, Jerome Dumortier, Nicolas de Prost, for the GrrrOH and Gref2 study groups, Guillaume VOIRIOT, Julien SCHMIDT, Jean Pierre QUENOT, Maxens DECAVELE, uliette AUDIBERT, Emmanuel CANET, Fabrice UHEL, Olivier LESIEUR, Romy YOUNAN, Anne Sophie MOREAU, Audrey DE JOND, Djamel MOKART, Mathilde NEUVILLE, Elie AZOULAY, Giuliana AMADDEO, Paer-Selim ABBACK, Charlotte BOUZBIB, Claire VANLEMMENS, Jerome DUMORTIER, Sylvie RADENNE, Armand ABERGEL, Céline GUICHON, Olivier ROUX, Agnes BONADONA, Nicolas CARBONELL, Christine SILVAIN, Helene MONTIALOUX, Jose URSIC

**Affiliations:** Assistance Publique—Hôpitaux de Paris, Hôpitaux Universitaires Henri Mondor, DHU ATVB, Service de Médecine Intensive Réanimation,Créteil, France; Université Paris Est Créteil, Faculté de Médecine de Créteil, Institut Mondor de Recherche Biomédicale—Groupe de Recherche Clinique CARMAS,Créteil, France; Assistance Publique—Hôpitaux de Paris, Hôpitaux Universitaires Henri Mondor, Service d’Hépatologie,Créteil, France; Sorbonne Université, INSERM, UMRS1158 Neurophysiologie Respiratoire Expérimentale et Clinique,Paris, France; Groupe Hospitalier Universitaire APHP-Sorbonne Université, Site Pitié-Salpêtrière, Service de Médecine Intensive et Réanimation (Département R3S),Paris, France; Département Anesthésie Réanimation, Université de Tours, CHU de Tours, Tours, France; Assistance Publique—Hôpitaux de Paris, Hôpitaux Universitaires Henri Mondor, DHU ATVB, Service de Médecine Intensive Réanimation,Créteil, France; Université Paris Est Créteil, Faculté de Médecine de Créteil, Institut Mondor de Recherche Biomédicale—Groupe de Recherche Clinique CARMAS,Créteil, France; Assistance Publique—Hôpitaux de Paris, Hôpitaux Universitaires Pitié Salpêtrière, Service d’Hépatologie,Paris, France; Centre Hospitalier Universitaire Jean Minjoz, Service d’Hépatologie et Soins Intensifs Digestifs,Besançon, France; Assistance Publique—Hôpitaux de Paris, Hôpital Universitaire Saint Louis, Service de Médecine Intensive Réanimation,Paris, France; Service de Médecine Intensive Réanimation, Centre Hospitalier Universitaire de Nantes, Nantes, France; Université de Nantes, Nantes, France; Centre Hospitalier Universitaire de Lille, Service de Réanimation Médicale,Lille, France; Hôpital Foch, Service de Réanimation Polyvalente,Suresnes, France; Assistance Publique—Hôpitaux de Paris, Hôpital Universitaire Saint Louis, Service de Médecine Intensive Réanimation,Paris, France; Assistance Publique—Hôpitaux de Paris, Hôpitaux Universitaires Pitié Salpêtrière, Service de Réanimation Chirurgicale,Paris, France; Institut Paoli-Calmettes, Service de Réanimation Polyvalente,Marseille, France; Hospices Civils de Lyon, Hôpital de la Croix-Rousse, Service d’Hépato-Gastroentérologie,Lyon, France; Centre Hospitalier Universitaire de Clermont-Ferrand, Service d’Hépato-Gastroentérologie,Clermont-Ferrand, France; Hospices Civils de Lyon, Hôpital de la Croix-Rousse, Service d’Anesthésie-Réanimation,Lyon, France; Assistance Publique—Hôpitaux de Paris, Hôpital Beaujon, Service d’Hépatologie,Clichy, France; Service d’Hépatologie et Greffe, Pôle Digidune, CHU Grenoble Alpes,La Tronche, France; Assistance Publique—Hôpitaux de Paris, Hôpitaux Universitaires Henri Mondor, DHU ATVB, Service de Médecine Intensive Réanimation,Créteil, France; Université Paris Est Créteil, Faculté de Médecine de Créteil, Institut Mondor de Recherche Biomédicale—Groupe de Recherche Clinique CARMAS,Créteil, France; PhyMedExp, Montpellier University, INSERM, CNRS, CHU de Montpellier, Montpellier, France; Department of Anesthesia and Intensive Care Unit, St-Eloi Hospital, Montpellier, France; Hospices Civils de Lyon, Hôpital Edouard Herriot, Service d’Hépato-Gastroentérologie,Lyon, France; Université Claude Bernard Lyon 1, Villeurbanne, France; Assistance Publique—Hôpitaux de Paris, Hôpitaux Universitaires Henri Mondor, DHU ATVB, Service de Médecine Intensive Réanimation,Créteil, France; Université Paris Est Créteil, Faculté de Médecine de Créteil, Institut Mondor de Recherche Biomédicale—Groupe de Recherche Clinique CARMAS,Créteil, France

**Keywords:** ICU, acyclovir, acute liver failure, hemophagocytic syndrome, herpes simplex virus

## Abstract

The clinical features and short-term prognosis of patients admitted to the intensive care unit for herpes hepatitis are lacking. Of 33 patients admitted between 2006 and 2022, 22 were immunocompromised, 4 were pregnant women, and 23 died. Sixteen patients developed a hemophagocytic syndrome. Acyclovir was initiated a median (interquartile range) of 1 (0–3) day after admission.

## Graphical Abstract

**Graphical Abstract:**
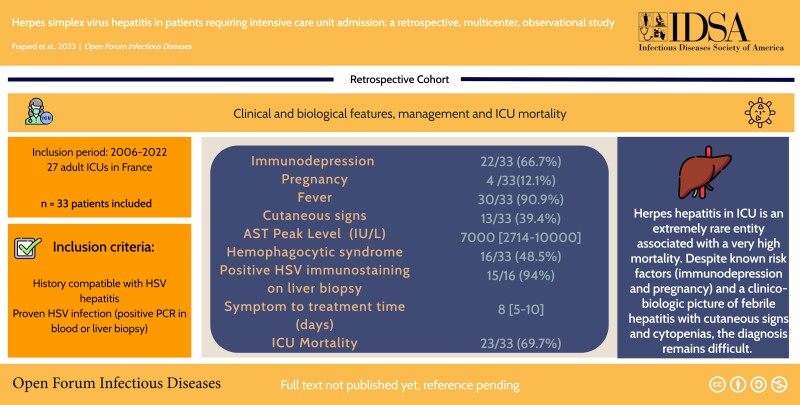
https://tidbitapp.io/tidbits/severe-herpes-simplex-virus-hepatitis-a-retrospective-multicenter-observational-study

Acute liver failure is a rare but life-threatening critical illness [1, 2]. One of the least reported causes is severe herpes simplex virus (HSV) hepatitis (HH). Its pathophysiology is not fully understood. Although primary infection is a major cause of herpes hepatitis [3–6], a few cases of HH associated with HSV reactivation, defined by immunoglobulin G (IgG) serology positivity, have been reported in immunocompromised patients [7]. The prevalence of HH appears to be around 0.8% of cases of severe hepatitis [8, 9].

Most of the available data consist of case reports with literature reviews [7, 10]. The clinical picture of HH is a febrile hepatic insufficiency with cutaneous signs and marked serum transaminase and lactate dehydrogenase elevation occurring in immunocompromised or pregnant patients, and to a lesser extent in the postoperative setting [3–7, 9]. The prognosis of HH has been reported to be poor, with a mortality rate of up to 60%, compared with 19%–36% for other causes of severe acute hepatitis. A diagnostic delay has been frequently reported, with only 30% of patients receiving early antiviral treatment with acyclovir [9].

There are to our knowledge no previous studies describing the clinical characteristics, management, and short-term outcomes of patients admitted to the intensive care unit (ICU) for HH. We conducted a large multicenter retrospective cohort study in adult patients admitted to French ICUs for acute hepatitis and proven HSV infection and aimed at describing their clinical and biological features, management, and ICU mortality.

## METHODS

### Patients

We performed a 16-year multicenter retrospective observational cohort study in 27 adult ICUs located throughout metropolitan France. All consecutive patients hospitalized in participating ICUs between 2006 and 2022 with a compatible history and a proven HSV infection (positive polymerase chain reaction [PCR] in blood or liver biopsy) were included (Supplementary Method 1).

### Ethical Considerations

This observational, noninterventional analysis of medical records was approved by the Institutional Review Board of the French Intensive Care Society in December 2021 (CE SRLF 21-108).

### Collection of Data

Data pertaining to demographics, comorbidities, clinical examinations, laboratory findings, microbiological investigations, and therapeutic management at ICU admission and during ICU stay were collected. Duplex HSV PCR methods were used in most centers and were qualitative for half the patients. Quantitative assessment of circulating HSV DNA was provided when available and expressed as log DNA copy numbers/mL of serum. Simplified Acute Physiology Score II (SAPS II) and Sequential Organ Failure Assessment (SOFA) scores were computed using the worst values recorded within the first 24 hours of admission. Missing data were retrieved by queries to the investigators.

### Statistical Analysis

Quantitative data were expressed as mean (SD) or median (interquartile range [IQR]) and compared using a Student *t* test or nonparametric Mann-Whitney test, as appropriate. Qualitative data were compared by χ^2^ test or Fisher exact test. Factors associated with ICU mortality were analyzed by univariable logistic regression. The tests were 2-tailed, and a value of *P* < .05 was considered statistically significant. Statistical analysis was performed using the software R 4.2.2 (The R Foundation for Statistical Computing, Vienna, Austria).

### Control Group

A bicentric control group cohort of ICU acetaminophen hepatitis patients was assembled to explore the specificity of the features of hemophagocytic syndrome observed in patients with HH (Supplementary Method 2).

## RESULTS

Among the 27 participating ICUs, 15 identified a total of 33 patients during the study period.

### Clinical and Biological Features

The median age (IQR) was 55 (37–65) years, with 15 patients (45.4%) of male gender. Risk factors for HH commonly reported in the literature included 22 (66.7%) immunosuppressed patients and 4 (12.1%) pregnant patients (3 were in the third trimester and 1 in the second trimester) (Table 1). Upon ICU admission, 30 (90.9%) patients were febrile, with a median temperature (IQR) of 39.2°C (39.1°C–40.2°C). Skin signs were present in 13 (39.4%) patients, and 27 (81.8%) patients had an altered mental status. All patients had at least 1 organ failure on admission, with a median SOFA score (IQR) of 14 (10–17). Thrombocytopenia was almost constant, with a median platelet count on admission (IQR) of 50 (22–82) G/L and a median (IQR) prothrombin time (PT) of 38% (28%–50%). There was a marked transaminase elevation, which was more pronounced on aspartate transaminase (AST); median serum value [IQR], 2694 [932–6217] IU/L) than on alanine transaminase (ALT) (Table 1).

**Table 1.** Clinical and Biological Characteristics at Intensive Care Unit Admission and Outcomes in 33 Patients With Proven HSV Hepatitis

Clinical and Biological Features at ICU Admission	Median [IQR] or No. (%)
Demographics	…
Age, y	55 [37–65]
Males	15 (45)
BMI, kg/m^2^	27 [26–30]
SAPS 2	60 [40–71]
SOFA	14 [10–17]
Pregnancy	4 (12.1)
Smoking	3 (9.1)
Comorbidities	…
Diabetes mellitus	5 (15.2)
Cardiovascular disease	4 (12.1)
Chronic kidney disease	4 (12.1)
Chronic respiratory failure	2 (6.1)
Obesity	4 (12.1)
Immunodepression	22 (66.7)
Immunodepression features	…
Solid organ transplant	4 (12.1)
Solid cancer	2 (6.1)
Hematologic disease	7 (21.2)
HIV	2 (6.1)
Prolonged corticosteroid therapy	9 (27.3)
Immunosuppressant	12 (36.4)
Reason for ICU admission	…
Multivisceral failure	10 (30.3)
Acute liver dysfunction	12 (36.4)
Sepsis	6 (18.2)
Acute respiratory distress	3 (9.1)
Central nervous failure	1 (3.0)
Other	1 (3.0)
Clinical symptoms on ICU admission	…
Fever	30 (90.9)
Temperature on admission, °C	39.2 [39.1–40.2]
Skin signs	13 (39.4)
Vesicles	9 (27.3)
Rash	6 (18.2)
Confusion	27 (81.8)
Organ support upon ICU admission	…
Vasopressors	21 (63.6)
Mechanical ventilation	17 (51.5)
Renal replacement therapy	12 (36.4)
Laboratory data on ICU admission (worst value within 24 h)	…
Blood leukocytes, G/L	2.8 [1.5–6.1]
Blood platelets, G/L	50 [22–82]
Prothrombin rate, %	38 [28–50]
Factor V, %	36 [24–51]
Arterial lactate level, mmol/L	7.2 [3–15]
Serum bicarbonate level, mmol/L	15.0 [13.0–20.0]
Serum creatinine, µmol/L	190 [99–300]
Serum bilirubin, µmol/L	25 [20–46]
ALT, IU/L	1056 [523–1700]
AST, IU/L	2694 [932–6217]
PAL, IU/L	150 [64–256]
GGT, IU/L	137 [58–265]
LDH, IU/L	5000 [2574–9943]

Abbreviations: ALT, alanine transaminase; AST, aspartate transaminase; BMI, body mass index; GGT, gamma-glutamyltransferase; HSV, herpes simplex virus; ICU, intensive care unit; IQR, interquartile range; LDH, lactate dehydrogenase; PAL, alkaline phosphatase; SAPS 2, Simplified Acute Physiology Score, version II; SOFA, Sepsis-related Organ Failure Assessment.

HH diagnosis was confirmed in all cases by a blood PCR, and a liver biopsy was performed in 16 (48.5%) patients, confirming diagnosis in all cases when performed (Table 2). When available, HSV IgG was negative for 94.1% of patients (n = 16/17; data missing for 16 patients) (Table 2).

**Table 2.** Clinical Findings and Hospital Outcomes in 33 Patients With Proven HSV Hepatitis

	Median [IQR] or No. (%)
Diagnosis	…
Positive blood PCR	33 (100)
HSV1	12/24 (50)
HSV2	12/24 (50)
N/A (serotype not available)	9
Viral load (14/33 patients), log DNA copies/mL	6.5 [5.2–8.0]
Liver biopsy obtained^a^	16 (48.5)
Negative HSV IgG when available	16/17 (94.1)
Clinical outcome during the stay in intensive care	…
Maximum temperature, °C	39.1 [38.6–40.0]
Hepatic encephalopathy	27 (82)
Proven HSV encephalitis	6 (18)
Myocarditis	4 (12.1)
Hemorrhagic shock	11 (33.3)
Hemophagocytic syndrome^b^	16 (48.5)
HScore (score)^c^	176 [104–240]
HScore (probability of HS), %	63 [1.9–98.9]
Disseminated intravascular coagulation	14 (42.4)
Antiviral treatment	…
Acyclovir initiated	32 (97)
Time from symptom to treatment, d	8 [5–10.3]
ICU admission to treatment, d	1 [0–3.3]
Biological evolution	…
AST peak level, IU/L	7000 [2714–10 000]
ALT peak level, IU/L	2000 [1100–4815]
PT nadir, %	20 [8–35]
Nadir factor V, %	17 [5–31]
Hemophagocytosis on myelogram, %	12 (41)
Outcomes	…
Mortality in ICU	23 (69.7)
30-d mortality	23 (69.7)
Liver transplant	4 (12.1)
Successful liver transplant	0/4 (0)

Abbreviations: ALT, alanine transaminase; AST, aspartate transaminase; HS, hemophagocytic syndrome; HSV, herpes simplex virus; ICU, intensive care unit; IQR, interquartile range; PT, prothrombin rate.

^a^Displaying massive hepatocyte necrosis (n = 16/16), viral inclusions (n = 8/16), and positive HSV immunostaining (n = 15/16).

^b^According to HLH 2004 criteria by Henter et al. [11].

^c^Score reported by Fardet et al. [12].

### Treatment

The vast majority of patients (n = 32/33, 97%) received acyclovir during their hospital stay (Table 2). The median time from symptom onset to initiation of acyclovir (IQR) was 8 (5–10) days, and the median time from ICU admission to initiation of acyclovir (IQR) was 1 (0–3) day.

Regarding organ support, 21 (63.6%) patients required vasopressors, 12 (36.4%) required renal replacement therapy, and 17 (51.5%) required invasive mechanical ventilation. Of the pregnant patients, 2 required emergency fetal extraction before ICU admission.

### Outcomes and Prevalence of Hemophagocytic Syndrome

During their ICU stay, 27 (82%) patients developed hepatic encephalopathy, 6 (18%) had proven HSV meningo-encephalitis, 11 (33.3%) developed hemorrhagic shock, and 14 (42.4%) developed disseminated intravascular coagulation (DIC). The serum AST level peaked (IQR) at 7000 (2714–10 000) IU/L, and the prothrombin time (PT) nadir (IQR) was 20% (8%–35%).

Unexpectedly, 16 (48.5%) patients developed hemophagocytic syndrome (HS) [13] during the course of their ICU stay. Hemophagocytosis was present on myelogram in 12 (41%) patients (Table 2). In order to establish whether HS was related to the severity of liver failure or a specific feature of severe HSV hepatitis, we compared patients from the current cohort with a control cohort of patients with severe acetaminophen hepatitis with regard to HS features (Supplementary Table 1). There were significantly fewer immunosuppressed patients and pregnant women in the acetaminophen hepatitis group than in the HH group (*P* < .001), with no fever (*P* < .001) and no cytopenia (*P* < .001). The HScore [12] was significantly lower, with no hemophagocytic syndrome diagnosed in the acetaminophen group, suggesting that HS was not related to the severity of liver failure itself.

Finally, 23 patients (69.7%) died in the intensive care unit. Four (12.1%) patients underwent unsuccessful liver transplantation and eventually died (Table 2).

### Factors Associated With ICU Death

Factors associated with mortality in univariable analysis included severity of illness scores (SAPS 2 and SOFA scores), an altered mental status and serum creatinine at ICU admission, and need for vasopressor support, occurrence of hepatic encephalopathy or DIC, and PT nadir during ICU stay (Supplementary Table 2).

## DISCUSSION

We describe the largest multicenter cohort of patients admitted to the ICU for herpetic hepatitis. The main findings of our study are as follows: (1) the 2 main causes of ICU admission were multivisceral and acute liver failure; (2) 67% of patients were immunocompromised and 12% were pregnant women; (3) clinical presentation was associated with frank fever in 91% of patients, cutaneous signs in 39% of patients, and marked transaminase elevation predominating on AST; and (4) ICU mortality was 70%.

As expected, the factors associated with ICU mortality included severity of illness scores and hepatic encephalopathy, which are indicative of liver and other organ failures. We found no statistically significant association between time to initiation of acyclovir and mortality. Nevertheless, the high mortality observed in our series suggests that clinicians should consider initiating empiric treatment with acyclovir in patients admitted for acute febrile hepatitis with a compatible clinical history. Importantly, studies comparing acyclovir with placebo in mechanically ventilated, critically ill patients have reported no statistically significant excess risk of renal failure [14]. For the most severe patients, liver transplantation may be considered [9]. However, in our series, only 4 patients required transplantation, and, regrettably, they eventually died.

As previously reported [11, 15], there was a significant proportion (48.5%) of patients developing hemophagocytic syndrome, confirmed on myelogram in most cases, during the course of ICU stay. In our series, the diagnosis was made on the basis of severe fever, almost constant immunosuppression, profound thrombocytopenia, hepatic abnormalities, and coagulation disorders (eg, DIC; 42.4%). Hemophagocytic syndrome in a patient at risk is a supplemental feature that should lead clinicians to suspect herpetic hepatitis.

Our study certainly has limitations, related to its retrospective and observational design and the long duration of the inclusion period, required by the rareness of HH.

The strengths of this study lie in its national multicenter design, involving liver and general ICUs throughout France. The relatively high number of cases, considering the rarity of the disease, makes it the largest worldwide series to our knowledge. The number of cases with histologic evidence (n = 16/33, 48.5%) also limits the subjectivity of the diagnosis.

Among the perspectives of this work, the delay in diagnosis leads to a reflection on diagnostic criteria. Whether patients with negative HSV PCR may have been missed in the current series cannot be excluded and raises the question of the best diagnostic and therapeutic strategy in this setting (eg, repeated PCR testing and empiric acyclovir initiation). Given the extreme rarity of this disease, national prospective registries would probably be needed to validate diagnostic criteria, reassess the prevalence of the disease, and evaluate more aggressive therapeutic strategies.

## CONCLUSIONS

HH in patients admitted to the ICU is an extremely rare entity associated with very high mortality. Despite known risk factors (immunodepression and pregnancy) and a clinico-biologic picture of febrile hepatitis, characterized by a marked transaminase elevation predominating on AST, with cutaneous signs and cytopenias, diagnosis remains difficult. Early acyclovir treatment is recommended.

## Supplementary Material

ofad484_Supplementary_Data

## Supplementary Data

Supplementary materials are available at *Open Forum Infectious Diseases* online. Consisting of data provided by the authors to benefit the reader, the posted materials are not copyedited and are the sole responsibility of the authors, so questions or comments should be addressed to the corresponding author.
