# Supplementary material for: Herpes Simplex Virus Hepatitis in Patients Requiring Intensive Care Unit Admission: A Retrospective, Multicenter, Observational Study
Source: Open Forum Infect Dis. 2023 Oct 3;10(11):ofad484. doi: 10.1093/ofid/ofad484 (PMC10629341; doi:10.1093/ofid/ofad484)
Supplement: ofad484_Supplementary_Data [file ofad484_supplementary_data.docx]

Supplementary material

Herpes simplex virus hepatitis in patients requiring intensive care unit admission: a retrospective, multicenter, observational study

Thomas FRAPARD, MD, Giuliana AMADDEO, MD PhD, Maxens DECAVELE, MD, Paer-Selim ABBACK, MD, Antoine GAILLET, MD, Charlotte BOUZBIB MD, Claire VANLEMMENS MD, Romy YOUNAN MD, Emmanuel CANET MD PhD, Anne Sophie MOREAU MD, Mathilde NEUVILLE MD, Elie AZOULAY MD PhD, Alexandre SITBON MD, Djamel MOKART MD PhD, Sylvie RADENNE MD, Armand ABERGEL MD PhD, Céline GUICHON MD, Olivier ROUX MD, Agnes BONADONA MD, Armand Mekontso Dessap MD PhD, Audrey De Jong MD PhD, Jerome Dumortier, MD PhD, Nicolas de Prost, MD PhD, for the GrrrOH and Gref2 study groups

**Table of content**

**Additional methods**…………………………………….…………………………………………………………………………………….2

**Table S1**……………………………………………………………………………………………………………………………………………..3

**Table S2**……………………………………………………………………………………………………………………………………………..4

**Supplementary method S1:** identifying patients

The investigator of each participating center was responsible for identifying patients, either from hospital medical reports, using the function “research the files in which the words ‘herpes simplex virus hepatitis ‘ occur” in Microsoft Windows, or through a search using the following International Classification of Diseases (10^th^ revision) codes: B00 (herpes virus), K72 (acute liver failure). The hospital discharge reports for all identified patients were anonymized and then electronically or conventionally mailed to the main investigator (T. F.). Clinical charts were reviewed to verify the inclusion criteria.

**Supplementary method S2:** control group

A bicentric control group cohort of ICU acetaminophen hepatitis was assembled through a search using the following International Classification of Diseases (10^th^ Revision) code: T391 (acetaminophen poisoning). The same data were collected

**Table S1:** Demographic, Clinical and Biological Characteristics at Intensive Care Unit Admission in 33 Patients With Proven HSV Hepatitis (HH) versus 19 Patients With Acetaminophen Hepatitis (AH)

| **Median [IQR] or N (%)** | **ICU-HH**  **(n = 33)** | **ICU-AH**  **(n = 19)** | **P** |
| --- | --- | --- | --- |
| Age, years  Males, n (%)  BMI, kg/m2  SAPS 2  SOFA  Pregnancy, n (%)  Immunodepression  Temperature (°C)  Leukocytes (G/L)  Platelets (G/L)  Haemoglobin (g/dl)  Prothrombin rate (%)  DIC  HS  -HScore (score)  -HScore (probability %)  Ferritin (µg/l, n=8) | 55 [36-65]  15 (45)  27 [26-30]  60 [38-71]  14 [9.7-17]  4 (12.1)  22 (66.7)  39.1 [38.6-40.0]  2.7 [1.4-6.2]  50 [20-83]  8.1 [7.1-10.6]  36.5 [27.8-50.5]  14 (42.4)  16 (48.5)  180 [104-240]  68.7 [1.9-98.9]  57084 [26500-102841] | 41 [31-58]  4 (21)  19 [18-21]  32 [22-46]  5 [4-8]  0 (0)  1 (5.3)  36.8 [36-37]  13 [9.7-18.0]  230 [189-304]  13 [11.1-13.0]  49 [31.5-73.3]  1 (5.3)  0 (0)  0.1 [0.0-9]  0.1 [0.00-0.001]  N/A | 0.135  0.205  **0.032**  **0.001**  **0.001**  0.283  **<0.001**  **<0.001**  **<0.001**  **<0.001**  **<0.001**  0.106  0.455  **<0.001**  **<0.001**  **<0.001**  **N/A** |

BMI, Body Mass Index ; SAPS II, Simplified Acute Physiology Score version II; SOFA, Sepsis-related Organ Failure Assessmen; HS, Hemophagocytic syndrome; DIC Diffuse Intravascular Coagulation

**Table S2.** Identification of factors associated with ICU death by univariable logistic regression in 33 patients with proven HSV hepatitis

| **Median [IQR] or N (%)** | **Survivors**  **(n = 10)** | **Non survivors**  **(n = 23)** | **OR [95%IC]** | **P** |
| --- | --- | --- | --- | --- |
| Age, years  Males, n (%)  BMI, kg/m2  SAPS 2  SOFA  Pregnancy, n (%)  Smoking, n (%)  Diabetes mellitus  Immunodepression  **Symptoms upon ICU admission**  Temperature on admission (°C)  Skin signs  Confusion  **Biology on ICU admission**  Leukocytes (G/L)  Platelets (G/L)  PT (%)  Factor V (%)  Lactate (mmol/L)  ALT (IU/L)  AST (IU/L)  Creatinine (µmol/L)  **Organ support on ICU admission**  Vasopressor  Mechanical ventilation  Renal replacement therapy  **HSV serotype**  -HSV1  -HSV2  -N/A  **Clinical evolution**  Hepatic encephalopathy  HSV encephalitis  Myocarditis  Hemorrhagic shock  HS  -HScore (score)  -HScore (probability %)  DIC  Time from symptom to treatment (day)  Hospitalization to  Treatment time (day)  ICU admission to treatment time (day)  **Biological evolution**  Peak AST (IU/L)  Peak ALT (IU/L)  Nadir TP (%)  Nadir factor V (%)  Liver transplantation | 45 [31-63]  3 (30)  27.0 [25.4-29.6]  44 [23-57]  9 [6-12]  2 (20)  0 (0)  1 (10)  6 (60)  39.1 [39-39.5]  4 (40)  6 (60.0)  3.1 [2.80-6.65]  84.00 [38-145.0]  38.50 [31.7-50.5]  41.50 [29.2-59.5]  2.95 [2.05-6.28]  1140 [566.5-1647]  2000 [977.5-5900]  52 [44-101]  4 (40)  5 (50)  3 (30)  1(10)  6 (60)  3 (30)  5 (50)  4 (40)  1 (10)  1 (10)  5 (50)  180 [127-219]  68.7 [8-96.2]  1 (10)  8. [7.25-9.00]  4.50 [1.75-6.75]  0.00 [0.00-1.75]  2607[1625-5625]  1200 [615-1925]  30.00 [30-40]  30 [25.25- 45]  0 (0.0) | 55 [41-65]  12 (52.2)  26.8 [26.3-30.2]  64 [55-73]  16 [12-17]  2 (8.7)  3 (13)  4 (17.4)  16 (69.6)  39.50 [37.5-40.0]  9 (39.1)  21 (93.3)  2.00 [1.20-3.90]  48.00 [20.0- 52.0]  38.00 [26.0-50.0]  29.50 [17.75-48.0]  11.2 [6.08-15.75]  1056 [520.5-1742]  2998 [1099-6768]  259 [169.5-343.5]  17 (73.9)  12 (52.2)  9 (39.1)  11 (47))  6 (26)  6 (26)  22 (95.7)  2 (8.7)  3 (13)  10 (43.5)  11 (47.8)  175 [102-241]  61.3 [1.80-99.1]  13 (56.4)  7.50 [5.00-12.00]  3.50 [1.25-13.00]  1.00 [0.00-3.75]  9999[7000-10974]  3250 [2000-5000]  10.00 [5.0-20.0]  10.50 [5.0-19.5]  4 (17.4) | 1.01 [0.98-1.07]  2.55 [0.55-14.68]  1.06 [0.86-1.32]  1.05 [1.01-1.11]  1.30 [1.07-1.62]  0.38 [0.03-3.20]  N/A  1.89 [0.24-39.9]  1.52 [0.36-9.18]  0.66 [0.10-1.91]  0.96 [0.17-4.12]  7.00 [1.28-60.5]  0.99 [0.89-1.09]  0.99 [0.98-1.00]  0.99 [0.95-1.03]  0.99 [0.96-1.02]  1.16 [0.95-1.58]  1.00 [1.00-1.00]  1.00 [1.00-1.00]  1.01 [1.00-1.02]  4.25 [1.13-22.49]  1.50 [0.18-4.42]  1.29 [0.19-6.12]  0.09 [0.01-0.70]  22.0 [2.25-388.3]  0.14 [0.01-0.91]  1.17 [0.12-25.82]  6.92 [1.04-138]  0.73 [0.11-3.42]  1.00 [0.99-1.01]  1.00 [0.98-1.02]  10.4 [1.5-209.5]  1.05 [0.92-1.31]  1.11 [0.99-1.38]  1.38 [0.93-2.43]  1.00 [1.00-1.00]  1.00 [1.00-1.00]  0.93 [0.87-0.98]  0.95 [0.91-1.00]  N/A | 0.520  0.247  0.670  **0.027**  **0.012**  0.314  N/A  0.591  0.593  0.509  0.983  **0.048**  0.724  0.088  0.492  0.340  0.204  0.469  0.446  **0.019**  **0.049**  0.863  0.976  0.093  **0.010**  **0.048**  0.901  0.088  0.625  0.869  0.846  **0.040**  0.480  0.224  0.284  **0.021**  0.149  **0.015**  **0.045**  N/A |

BMI, Body Mass Index ; ICU: Intensive care unit; SAPS II, Simplified Acute Physiology Score version II; SOFA, Sepsis-related Organ Failure Assessmen; SOT, Solid organ transplantation; HIV, Human immunodeficiency virus; CTC, Prolonged corticosteroid therapy; PT, Prothrombin rate; HS, Hemophagocytic syndrome; DIC Diffuse Intravascular Coagulation; **bolded** values are significant at the p<0.05 level.
